# Pulmonary veno-occlusive disease in Sjogren's syndrome: a case report

**DOI:** 10.1186/s12890-023-02322-w

**Published:** 2023-01-18

**Authors:** Xiaofang Zeng, Qiong Liu, Anandharajan Rathinasabapathy, Lihuang Zha, Dongliang Liu, Yiyang Tang, Jing Sun, Hui Luo, Zaixin Yu

**Affiliations:** 1 Department of Cardiology, Xiangya Hospital, Central South University, 87 Xiangya Road, Changsha, 410008 Hunan China; 2 National Clinical Research Center for Geriatric Disorders, Xiangya Hospital, Central South University, Changsha, Hunan China; 3 Division of Allergy, Pulmonary, and Critical Care Medicine, Vanderbilt University Medical Center, Nashville, TN USA; 4 Department of Emergency Medicine, Xiangya Hospital, Central South University, Changsha, Hunan China; 5 Department of Cardiology, The First Hospital of Changsha, Changsha, Hunan China

**Keywords:** pulmonary veno-occlusive disease, pulmonary arterial hypertension, Sjogren’s syndrome, *EIF2AK4*

## Abstract

**Background:**

Pulmonary arterial hypertension (PAH) associated with connective tissue disease (CTD) belongs to Group 1 pulmonary hypertension. Pulmonary veno-occlusive disease (PVOD), which is characterized by venous system aberrations, has been previously reported in CTD-PAH; however, it has rarely been observed in Sjogren’s syndrome (SS).

**Case presentation:**

Our 28-year-old female patient was admitted to the hospital with recurrent shortness of breath even after minimal physical activity. Her chest high-resolution CT scan demonstrated pulmonary artery dilatation and bilateral ground-glass nodules. A subsequent right heart catheterization confirmed pulmonary hypertension because her mean pulmonary arterial pressure was 62 mmHg. Our inquisitive genomic assessment identified a novel *EIF2AK4* mutation at c.1021 C > T (p. Gln341*), the dominant causal gene of PVOD. Histological examination demonstrated stenosis and occlusions in the pulmonary veins. Because she presented with features such as dry eyes and Raynaud's phenomenon, we performed a biopsy on the labial salivary gland, which confirmed SS. Her treatment regimen included PAH-targeted therapies (tadalafil and macitentan) in combination with hydroxychloroquine. Although she was hospitalized several times due to acute exacerbation of PAH, her disease progression was under control, and she did not demonstrate any signs of pulmonary edema even after a three-year treatment period.

**Conclusion:**

Here, we report the case of an SS-PAH patient with PVOD who carried a novel biallelic *EIF2AK4* mutation, and PAH-targeted therapies were well tolerated by our patient.

**Supplementary Information:**

The online version contains supplementary material available at 10.1186/s12890-023-02322-w.

## Background

Pulmonary hypertension (PH) is associated with connective tissue diseases (CTDs), such as systemic sclerosis (SSc), systemic lupus erythematosus, and Sjogren’s syndrome (SS) [1]. Because idiopathic PAH, pulmonary veno-occlusive disease (PVOD), left heart disease-associated PH and other pulmonary disorders could all drive the etiology of pulmonary hypertension (PH) in CTD patients [1, 2], it is natural that more than one etiological factor could coexist in accelerating the disease pathogenesis, and the primary etiological factor could even change during disease progression, complicating its management [3]. Hence, these etiological factors should be carefully evaluated since an inappropriate pharmacological intervention could accelerate the progression of CTD-PH.

In general, pulmonary vasodilator therapy does not elicit favorable responses in CTD patients with pulmonary arterial hypertension (CTD-PAH); thus, these patients have demonstrated poor prognosis compared with idiopathic PAH patients [1]. When CTD-PAH patients suffer from left heart disease, the pulmonary venous pressure is passively elevated due to an increased left-sided filling pressure, which overall resonates with the features of PVOD. In this condition, PAH therapies raise the risk of vasodilation mediated pulmonary edema for those patients instead of providing any potential benefits [4, 5].

PVOD belongs to Group 1 PH and is characterized by preferential remodeling of pulmonary venules [6]. Mutations in the eukaryotic translation initiation Factor 2 alpha kinase 4 (*EIF2AK4*) gene have been recently identified as the dominant cause of familial PVOD [7–9]. Incidentally, a biallelic *EIF2AK4* mutation is considered a standalone marker in the diagnosis of heritable PVOD [10, 11]. Clinically, vasodilators are considered with caution for the treatment of PVOD because of their association with life-threatening drug-induced pulmonary edema [12].

Because PVOD and PAH demonstrate overlapping clinical features, case presentations and examinations in PVOD patients are often unhelpful in distinguishing between these conditions [13]. Duarte et al*.* reported that previously diagnosed SSc-PAH patients developed acute pulmonary edema after the administration of sildenafil and bosentan, and in the follow-up exams, the diagnosis was changed to PVOD [14]. However, PVOD has rarely been reported in SS patients. Herein, we describe the interesting case of an SS-PAH patient who had PVOD and carried a novel biallelic *EIF2AK4* mutation at c.1021 C > T (p. Gln341*); we also noticed that PAH-targeted therapies were well tolerated by our patient and did not cause pulmonary edema after following the patient for a three-year follow-up period.

### Case presentation

Our 28-year-old female patient was admitted to the hospital with recurrent shortness of breath for one year, even after minimal physical activity, and the symptoms were aggravated for 3 days. She did not have a family history of either PH or any lung or heart diseases. She was a teetotaler, nonsmoker and never abused any addictive drugs or had other PVOD-associated risk factors [10]. Her preliminary examination demonstrated that she was afebrile and presented with the following physical examination parameters: 121/82 mmHg blood pressure, 101 beats/min heart rate, 22 breaths/min respiratory rate, 92% oxygen saturation index on room air, and a New York Heart Association functional class III heart failure. She had clear bilateral breathing sounds and an accentuated second pulmonic heart sound. Her bilateral lower limbs displayed slight pitting edema, while dermal examination demonstrated symptoms of Raynaud's phenomenon in both hands. Other laboratory values, such as thyroid function test, erythrocyte sedimentation rate, HIV, D-dimer, C reactive protein, rheumatic factor, antiphospholipid antibody, and anti-vasculitis antibody, were within the normal range.

**Fig. 1:**
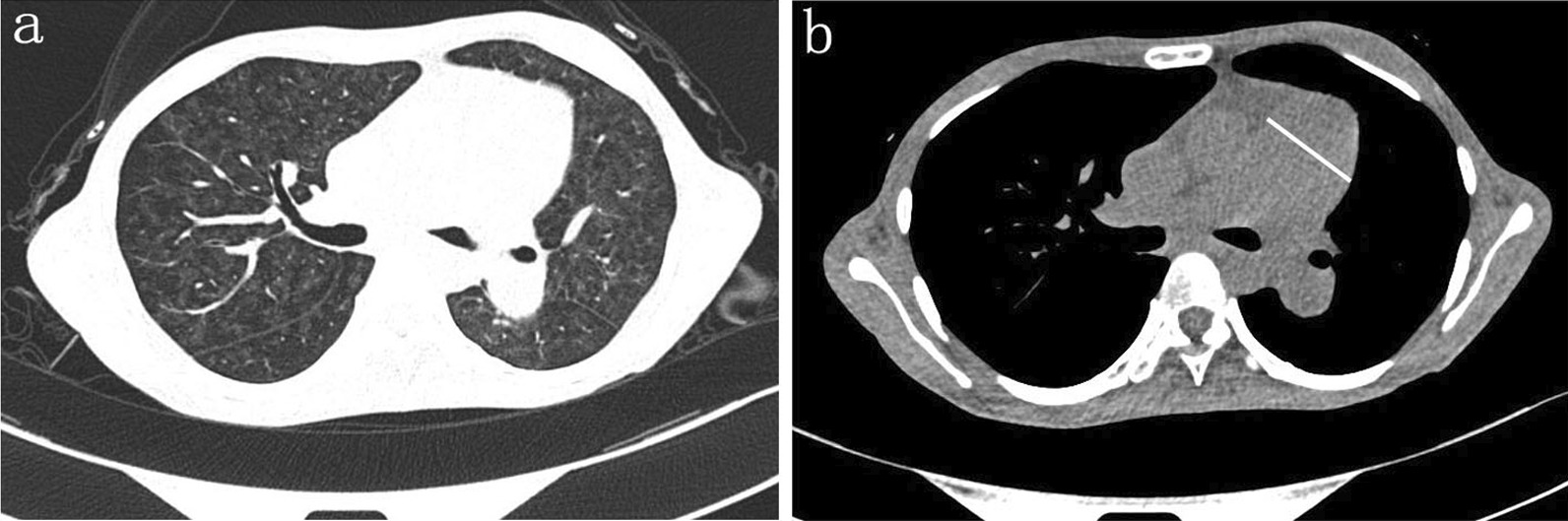
Representative chest high-resolution CT image revealing ground-glass opacities and a dilated pulmonary main trunk in a patient who has PVOD concurrent with Sjogren syndrome. The white line across the main pulmonary artery shows the dilated main pulmonary artery, with an estimated inner diameter of 38 mm

Her transthoracic echocardiogram illustrated PH features such as right ventricle hypertrophy, moderate tricuspid regurgitation, mild pericardial effusion, preserved left ventricular ejection fraction (62%) and fractional shortening (36%). A subsequent right heart catheterization at rest confirmed the diagnosis of PH: the patient had a pulmonary artery pressure of 62 mmHg, a pulmonary artery wedge pressure of 10 mmHg, a cardiac output by thermodilution of 2.05 L/min and a calculated pulmonary vascular resistance of 25 Wood units. Although the pulmonary ventilation/perfusion scan did not show any signs of thromboembolic disease, high-resolution computed tomography (HRCT) illustrated a dilated pulmonary artery, slight pericardial effusion and bilateral ground-glass nodules/opacities (Fig. 1). Her abdominal ultrasound demonstrated a crude and thick-walled gallbladder, hepatomegaly, seroperitoneum and an increased inner diameter of the inferior vena cava and hepatic vein, suggesting hepatic congestion, while the pulmonary function test showed moderate-to-severe mixed ventilation dysfunction.

Sjögren's syndrome (SS) is an autoimmune disease in which the glands of external secretions, such as the eye and mouth, are inflamed. Since our patient presented with symptoms of dry eyes and Raynaud's phenomenon, which were in combination with being positive for anti-nuclear antibody (anti-SSA, + +) and having increased level of procalcitonin, a labial salivary gland biopsy was performed that demonstrated the infiltration of lymphocyte foci in between the salivary glands—a valid confirmation of the diagnosis of SS.

**Fig. 2:**
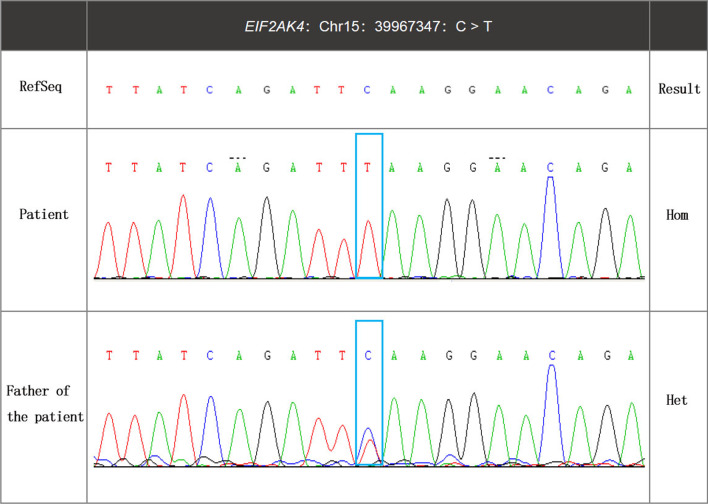
Representative electropherogram of Sanger sequencing identified an EIF2AK4 homozygous mutation at c.1021 C > T (p. Gln341*) in the patient and a heterozygous mutation in the patient’s father

**Fig. 3:**
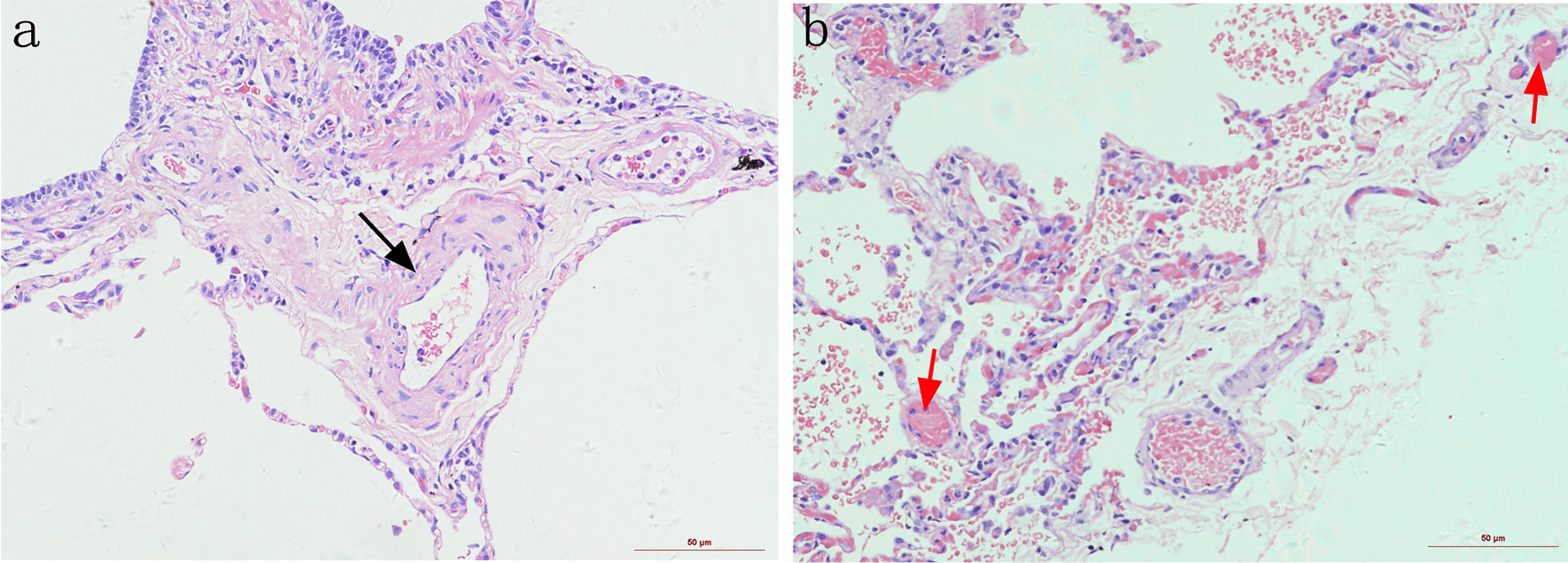
Representative hematoxylin & eosin staining displaying venule thickening and luminal occlusion (red arrow) and small pulmonary artery pronounced proliferation (black arrow)

Because our patient demonstrated bilateral ground-glass nodules/opacities, we did not rule out the possibility of PVOD. Hence, whole exome sequencing was carried out, and we identified a biallelic mutation in *EIF2AK4* at c.1021 C > T (p. Gln341*) (Table 1). In addition, pedigree screening led us to identify a heterozygous *EIF2AK4* mutation in her father’s DNA (Fig. 2). Furthermore, her thoracoscopic biopsy demonstrated marked venule thickening and luminal occlusion due to intimal proliferation and medial hypertrophy (Fig. 3), validating the diagnosis of PVOD. After multidisciplinary discussion and being fully informed about the risks to the patient, PAH-targeted therapies, such as tadalafil (20 mg, qd), macitentan (10 mg, qd), hydroxychloroquine (0.2 g, qd), and other symptomatic treatments, were initiated. The timeline of disease process for this patient was shown in Additional file 1. Her therapeutic regimens were continued for the next three years with periodic follow-up. Although she was admitted to the hospital multiple times for acute exacerbation of PAH (Table 2) during her three-year treatment period, surprisingly, she showed a satisfactory clinical response to PAH therapies, her disease symptoms were alleviated, and no sign of pulmonary edema was observed during the follow-up period.

**Table 1:** Genetic information of the patient from whole exosome sequencing

Gene	Chromosomal location	Variant	Mutant type	Pathogenicity classification	Genetic mode	Disease/phenotype
EIF2AK4	Chr15: 39967347	NM_001013703.4:exon9: c.1021C > T (p.Gln341*)	Hom	pathogenic	AR	PVOD

AR autosomal recessive, Hom homozygosis, PVOD pulmonary veno-occlusive disease,

A Variant name followed Human Genome Variation Society nomenclature (http://varnomen.hgvs.org/)

**Table 2:** Summarized treatment course of the patient

Time of admission	2018.11	2019.3	2021.6
RVSP^a^ (mmHg)	60	69	78
PA^a^(mm)	27	30	30
EF^a^ (%)	62	53	52
TAPSE ^a^(mm)	17	12	10
RV ^a^ (mm)	48	53	60
NT-proBNP(ng/ml, pre-treatment)	2482	2572	3445
NT-proBNP(ng/ml, post-treatment)	780	650	846
Functional class (NYHA, pre-treatment)	III	III	III
Functional class (NYHA, post-treatment)	II	II	II
PAH-target drugs	Tadalafil (10 mg,qd)	Tadalafil (20 mg,qd) Macitentan(10 mg,qd)	Tadalafil (20 mg,qd) Macitentan(10 mg,qd)

^a^based echocardiography; EF was measured for the left ventricle; RV were determined by subxiphoid four chamber section; PA were measured by short axis section of aorta; EF: ejection function; NYHA: New York Heart Association; NT-proBNP N-terminal pro-brain natriuretic peptide; PA: pulmonary artery; PAH: pulmonary artery hypertension; RV: right ventricular; RVSP: right ventricular systolic pressure; TAPSE: Tricuspid annular plane systolic excursion

## Discussion and conclusions

Characterized by specific diffuse occlusion of veins, PVOD has a worse prognosis than PAH [6, 15] Apart from arterial lesions, occlusive venopathy may occur in CTD-PAH diseases such as SSc-PAH [16, 17]. Previous studies have suggested that CTD-PAH is highly resistant to PAH-specific therapy, and hence, its prognosis is much worse than that of other forms of PAH [16, 18, 19]. Plausibly, this could, in part, because PVOD is highly prevalent in CTD patients, especially in those undergoing lung transplantation; hence, PVOD may serve as an unrecognized contributor to the dismal prognosis of these patients [20]. Overall, these clinical observations suggest that the coexistence of lesions in both small pulmonary veins and arteries in CTD could be the primary factor driving a less favorable response to therapeutic intervention and poor prognosis in CTD-PAH patients [21].

PVOD and PAH share numerous similarities, and most PVOD patients are prescribed PAH-specific vasodilator therapy whenever a definite clinical or radiological assessment is not established [3]. Since the obstruction of blood vessels in PVOD is primarily observed in the venous section, a vasodilator therapy that relaxes precapillary rather than postcapillary vessels often leads to blood flow-induced pulmonary edema in the postcapillary vascular bed instead of treating the disease [2]. Duarte et al. [14] reported an observation where the SSc-associated PVOD patient developed pulmonary edema after the initiation of vasodilator therapies. However, our patient demonstrated a good response to vasodilator therapies and did not present with pulmonary edema during her three-year drug regimen. Retrospectively, these outcomes suggest heterogeneity in vasodilator-mediated therapies for the treatment of PVOD.

*EIF2AK4 (*dominant causal gene of familial PVOD), which encodes kinase ‘general control nonderepressable 2′ (GCN2), plays a key role in maintaining essential cellular processes such as nutrient starvation and oxidative stress [22]. *EIF2AK4* mutation carriers were characterized by severe intimal fibrosis and lower GCN2 expression than *EIF2AK4* noncarriers, suggesting an association between GCN2 and disease phenotype [23]. GCN2 is activated by binding of uncharged tRNA to its histidyl-tRNA synthetase (HisRS)-like domain. The degenerate kinase domain interacts with translating ribosomes and may facilitate the transfer of uncharged tRNA from the ribosomal decoding site to the HisRS domain [24, 25]. In our patient, the EIF2AK4 mutation (c.1021 C > T (p. Gln341*) is located at the degenerate kinase domain and causes a shift from cytosine to thymine, which introduces a premature stop codon at the 341st glutamine. This premature stop would at least moderately disrupt the synthesis of GCN2 proteins.

In a three-year follow-up study, Li et al. reported that PAH-targeted therapies were well tolerated in a PVOD patient with a biallelic *EIF2AK4* mutation at c.1392delT (p.Arg465fs) [26]. Later, Zhang et al. [27] also reported a similar response to PAH-targeted therapies in two different PVOD patients who carried biallelic *EIF2AK4* mutations at c.1387delT (p. Arg463fs) or c.989–990 delAA (p. Lys330fs). Similar to these observations, PAH-targeted therapies were well tolerated by our patient and did not cause pulmonary edema during the three-year treatment period. However, Tenorio et al. reported that the *EIF2AK4* mutation c.3344C > T (p. P1115 L) caused an early onset of disease and low survival rate in six consanguineous PVOD patients [8]. Another report of a case of an *EIF2AK4* mutation at c.4833_4836dup (p. Q1613Kfs*10), reported that this mutation caused an aggressive phenotype, such that the time from onset of disease symptoms to death was less than 6 months [28]. We are surprised to note that all of those patients who responded well to PAH-targeted therapies had the *EIF2AK4* mutation located in the degenerate kinase-like domain (amino acids between 280 and 537), while the aggressive phenotype mutation is located at the HisRS domain (amino acids between 1021 and 1492) or C-terminal (amino acids between 1492 and 1648) [24, 25]. Taken together, we hypothesize that the location of *EIF2AK4* mutation could primarily drive this heterogenic therapeutic response, thus leading to a differential disease phenotype, warranting a detailed follow-up.

To summarize, our case study is the first report to present a patient with PVOD-associated SS-PAH, who carried a novel *EIF2AK4* mutation and who responded well to PAH-targeted therapies. We also conclude that the location of *EIF2AK4* mutation could be one of the major factors responsible for the different responses to targeted therapy and different disease phenotypes.

## Supplementary Information

**Additional file 1**. The Timeline of disease process of this patient.

## Data Availability

The datasets generated during the current study are available in the in the Genome Sequence Archive (Genomics, Proteomics & Bioinformatics 2021) in National Genomics Data Center (Nucleic Acids Res 2022), China National Center for Bioinformation/Beijing Institute of Genomics, Chinese Academy of Sciences (GSA-Human: HRA003715) that are publicly accessible at https://ngdc.cncb.ac.cn/gsa-human.

## Abbreviations

CTD: Connective tissue diseases
EIF2AK4: Eukaryotic translation initiation factor 2 alpha kinase 4
GN2: Control nonderepressable 2
HisRS: Histidyl-tRNA synthetase
HRCT: High resolution computed tomography
PAH: Pulmonary artery hypertension
PH: Pulmonary hypertension
PVOD: Pulmonary veno-occlusive disease
SS: Sjogren’s syndrome
SSc: Systemic sclerosis
