# Supplementary material for: Pulmonary veno-occlusive disease in Sjogren's syndrome: a case report
Source: BMC Pulm Med. 2023 Jan 18;23:26. doi: 10.1186/s12890-023-02322-w (PMC9847112; doi:10.1186/s12890-023-02322-w)
Supplement: Supplementary file 1 — Additional file 1. The Timeline of disease process of this patient. [file 12890_2023_2322_MOESM1_ESM.docx]

**Supplementary table: Timeline of disease process for this PVOD with SS-PAH patient**

| DATA | Relevant Past Medical History and Interventions | | |
| --- | --- | --- | --- |
| **In the past** | This patient did not present a family history of neither PH nor any lung or heart diseases. She was a teetotaler, non-smoker and never abused addictive drugs nor had other PVOD associated risk factors | | |
| DATA | **Summaries from Initial and Follow - up Visits** | **Diagnostic Testing**  **(including dates )** | **Interventions** |
| **20181114-20181201** | She was admitted in the hospital with repeated shortness of breath and lower limb edema after she gave a birth one year ago. Based on physical examination and related clinical examination, including RHC, HRCT, histological examination, and labial salivary gland biopsy, the diagnosis of PVOD concurrent with SS related PAH have been established. | Laboratory test;  Echocardiography;  Chest HRCT;  Labial salivary gland biopsy;  Histological examination;  Abdominal ultrasound;  Pulmonary function test | Oxygen inhalation;  Diuresis  Cardiotonic  Potassium supplement  Hydroxychloroquine  Tadalafil |
| **20190306-20190318** | She was admitted in the hospital for acute exacerbation of PAH symptoms | Laboratory test;  Echocardiography;  Chest HRCT;  Abdominal ultrasound; | Oxygen inhalation;  Diuresis  Cardiotonic  Potassium supplement  Hydroxychloroquine  Tadalafil  Macitentan |
| **20210624-20190702** | She was admitted in the hospital for acute exacerbation of PAH symptoms | Laboratory test;  Echocardiography;  Chest HRCT;  Abdominal ultrasound; | Oxygen inhalation;  Diuresis  Cardiotonic  Potassium supplement  Hydroxychloroquine  Tadalafil  Macitentan |
|  | Final outcome for this episode of care: This patient was hospitalized several times due to acute exacerbation of PAH; however, her disease progression was under control, and she did not demonstrate any signs of pulmonary edema following the three year treatment period. | | |

HRCT: High resolution computed tomography; PAH: Pulmonary artery hypertension; PH: Pulmonary hypertension; PVOD: Pulmonary veno-occlusive disease; SS: Sjogren’s syndrome;
